# Primary Cardiac Angiosarcoma Presenting as Cardiac Tamponade

**DOI:** 10.7759/cureus.29033

**Published:** 2022-09-11

**Authors:** Emad Elmusa, Muhammad W Raza, Hao Zhang, Naja Naddaf, Ahmad Muneeb

**Affiliations:** 1 Internal Medicine, Hospital Corporation of America Florida (HCA FL) Orange Park Hospital, Orange Park, USA

**Keywords:** pericardial effusion, primary cardiac tumor, cardiac tumor in adults, cardiac tamponade, primary cardiac angiosarcoma

## Abstract

Malignant primary cardiac tumors are rare. The most common presenting symptom is dyspnea, which is non-specific. These tumors pose a significant diagnostic challenge, which when coupled with rapid disease progression can result in significant morbidity and mortality. Appearance of cardiac masses on CT and echocardiography can be non-specific. Cardiac MRI can help delineate cardiac tumors but definitive diagnosis requires mediastinal exploration and biopsy. Treatment includes radical resection followed by radiotherapy and chemotherapy along with targeted therapy. Metastasis often precludes candidacy for surgery, therefore, early diagnosis is pivotal. We present a patient with primary cardiac angiosarcoma who initially presented with cardiac tamponade and at time of diagnosis was not a surgical candidate. We aim to bring greater awareness to malignant primary cardiac tumors in hopes of increasing diagnostic suspicion to facilitate earlier diagnosis and treatment intervention.

## Introduction

The majority of primary cardiac tumors are benign and most commonly occur in the left side of the heart [1]. A smaller percentage -- around 20% -- are malignant, and most commonly occur in the right side of the heart [2-3]. Cardiac MRI can help distinguish and differentiate cardiac masses but is not readily available. Definitive diagnosis requires surgical exploration and biopsy. With surgery, patients with primary cardiac angiosarcoma have a median survival of 14 months [4], while patients with metastasis who cannot undergo surgery have a median survival of approximately 3.8 +/- 2.5 months [1]. At the time of diagnosis, 89% of patients already have evidence of metastasis [1]. Thus, early diagnosis is critical to facilitate candidacy for surgery and reduce morbidity and mortality. We are presenting a case of a patient with primary cardiac angiosarcoma who was not a surgical candidate at the time of diagnosis, and who ultimately died from obstructive shock.

## Case presentation

**Figure 1:**
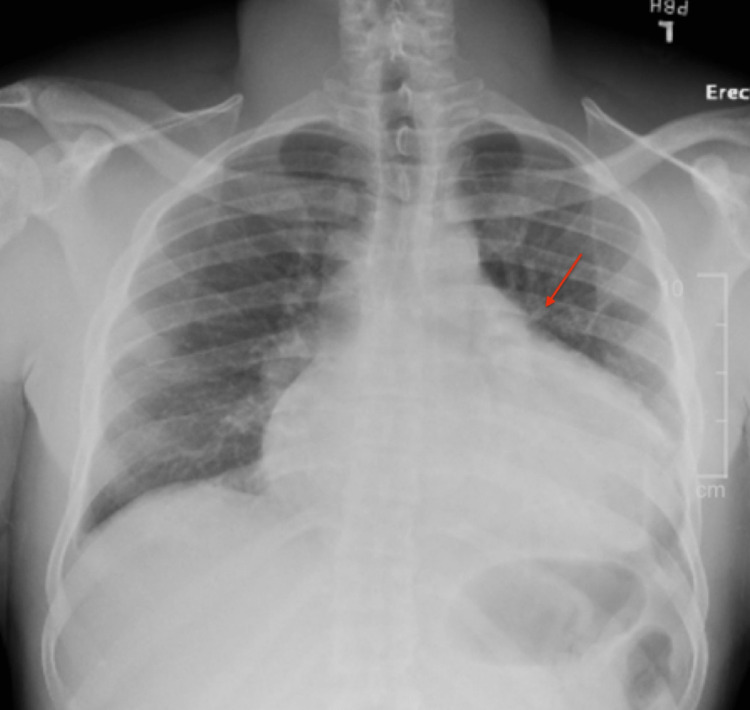
Chest X-ray demonstrating a widened mediastinum.

**Figure 2:**
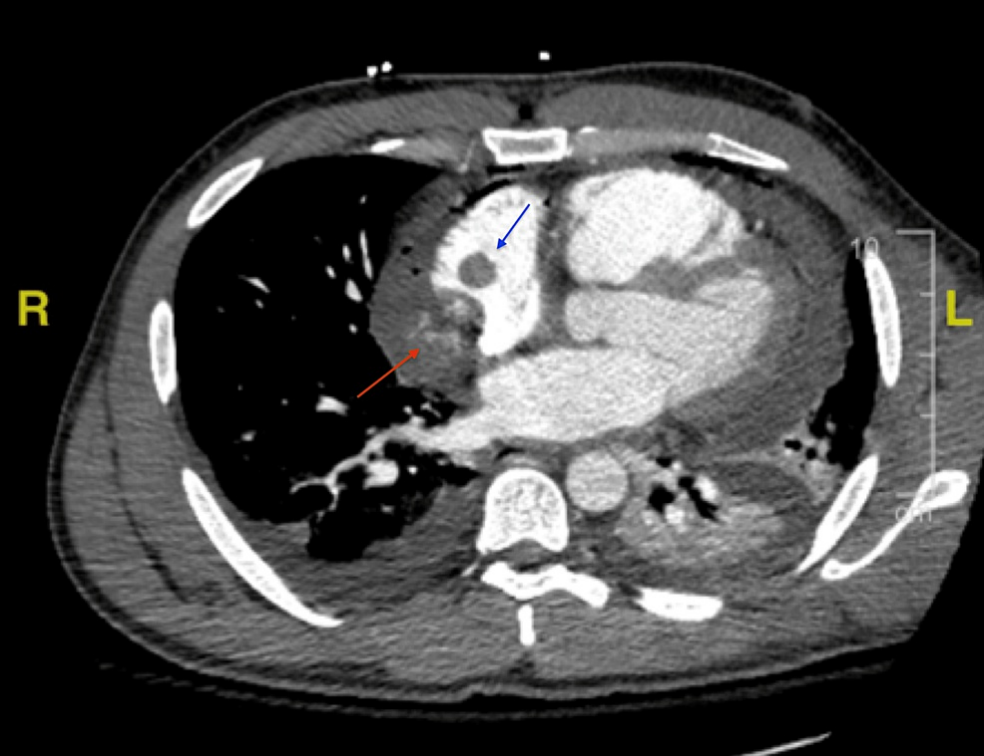
CTA chest depicting small amount of heterogeneous hyperdense material along the medial aspect of the right atrium (red arrow) and a 1.4 cm mass at the superior medial aspect of the right atrium (blue arrow). CTA, computed tomography angiogram

A 35-year-old African American male with no significant past medical history presented with a chief complaint of dyspnea on exertion that worsened over the past month. He denied history of tobacco use, alcohol dependence, prior malignancy, or family history of cancer. On presentation in the emergency department, vitals revealed a temperature of 98.2°F, heart rate of 118 bpm, respiratory rate of 17 bpm, blood pressure of 122/83 mmHg, and pulse oxygen saturation 99%. Labs were significant for high sensitivity cardiac troponin of 310 pg/mL (reference 3-45.20 pg/mL) and a hemoglobin of 11.9 g/dL (reference 11.2-15.7 g/dL). Chest radiography revealed widening of the mediastinum (Figure 1). Transthoracic echocardiogram revealed a large partially loculated pericardial effusion along the right atrial free wall with early right ventricle diastolic collapse suggestive of tamponade physiology. The patient was subsequently taken to the operating room for a pericardial window. As the pericardium was incised, there was immediate bloody fluid return, with a total of 1.2 L removed from the pericardial space. A transesophageal echocardiogram was performed intraoperatively, which revealed a large mass adhered to the right atrium measuring 6.5 cm with a 1.6 cm mobile mass attached to it. At this time, it was unclear if this was representative of a tumor, a thrombus, or both. The patient subsequently underwent computed tomography pulmonary angiography (CTPA) for evaluation of pulmonary embolism (PE) (Figure 2). CTPA was negative for PE, but did reveal a small amount of heterogeneous hyperdense material along the medial aspect of the right atrium concerning for active hemorrhage. Additionally, a 1.4 cm mass was seen at the superior medial aspect of the right atrium. Again, it was still not clear what the mass represented. The patient improved during the next three days, and hemoglobin remained stable. Pericardial biopsy was negative for malignancy. The patient was discharged home with apixaban, for concern the mass represented a thrombus. The plan was for the patient to have an outpatient cardiac MRI to delineate the intracardiac mass and to follow up with cardiology and cardiothoracic surgery in the outpatient setting.

 

**Figure 3:**
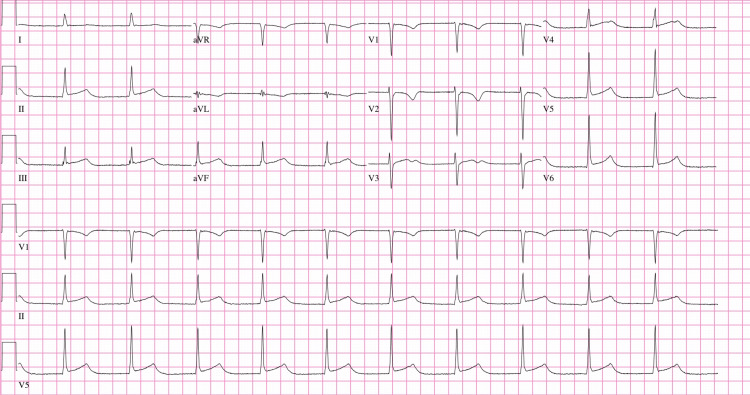
Electrocardiogram with diffuse ST changes.

**Figure 4:**
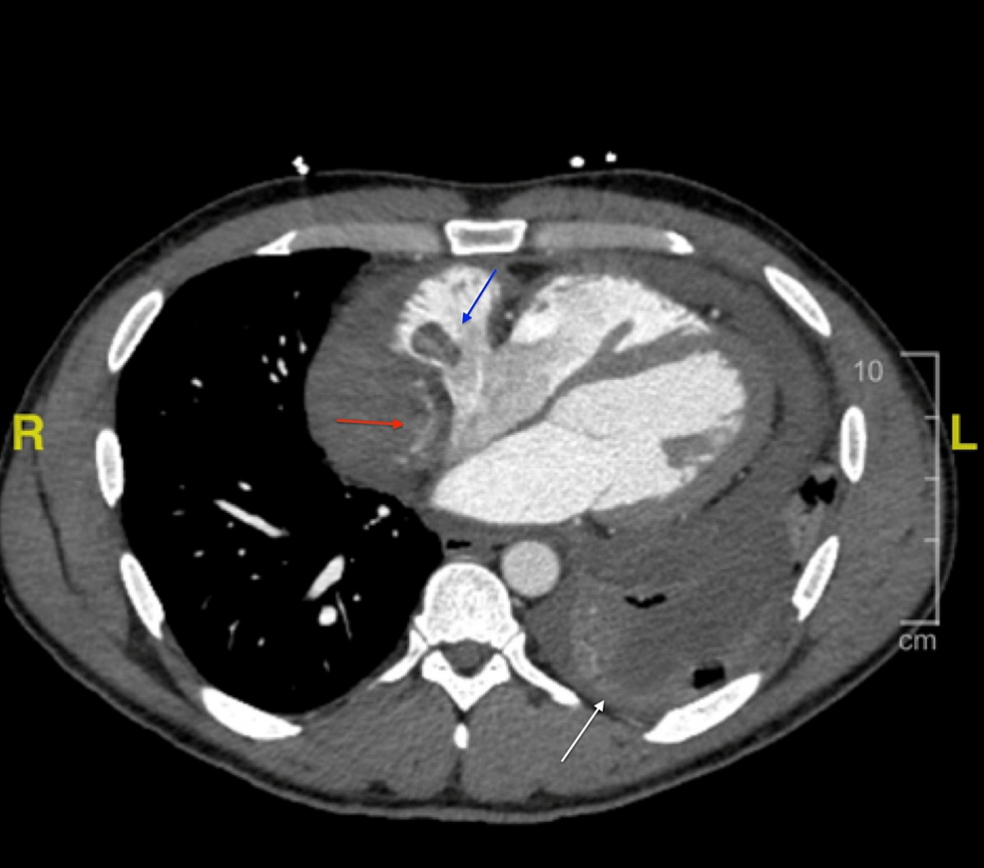
CT chest angiogram demonstrating complex hyperattenuating pericardial effusion, with contrast seen adjacent to the right (red arrow). A focal filling defect was also appreciated within the right atrium, measuring 1.9 cm (blue arrow). A moderate-sized left-sided pleural effusion was also noted (white arrow).

**Figure 5:**
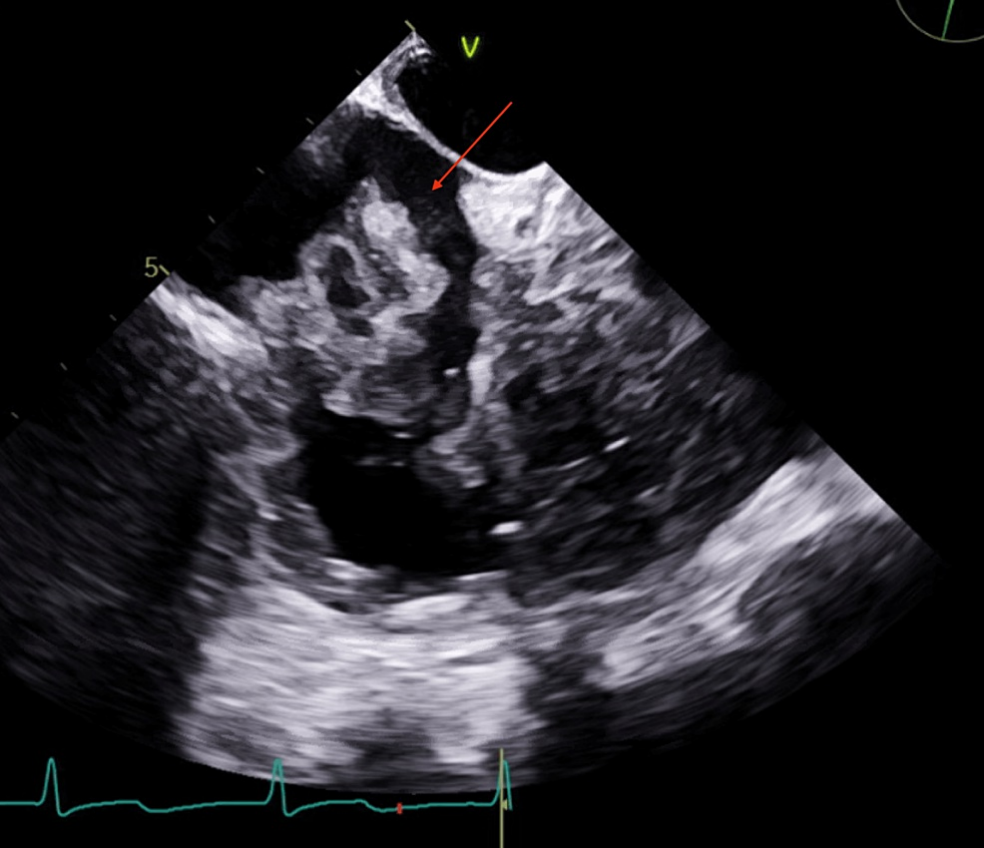
Transesophageal echocardiogram with intra right atrial mass with mobile attachments.

**Figure 6:**
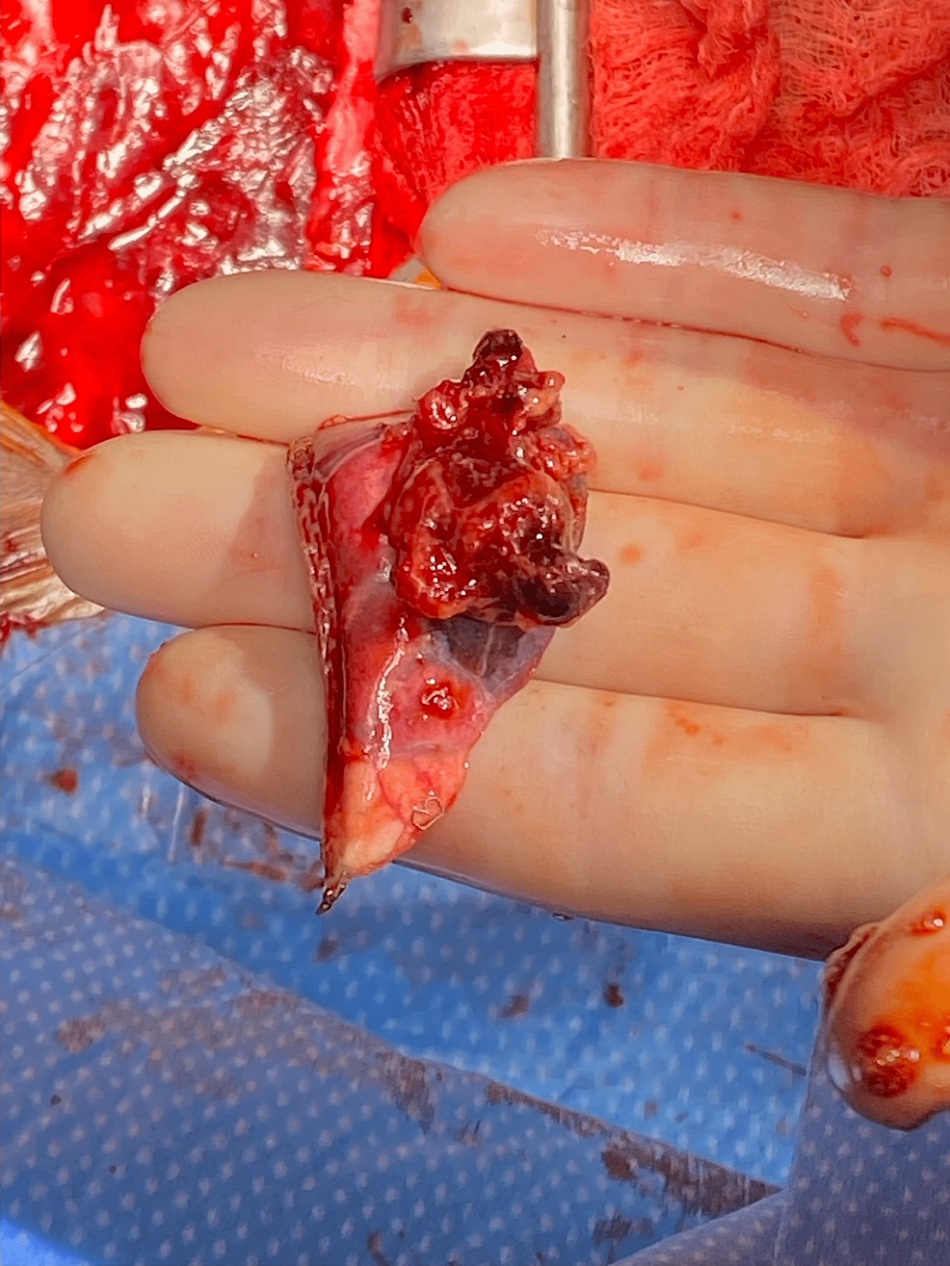
Intraoperative image showcasing right atrial mass biopsy.

**Figure 7:**
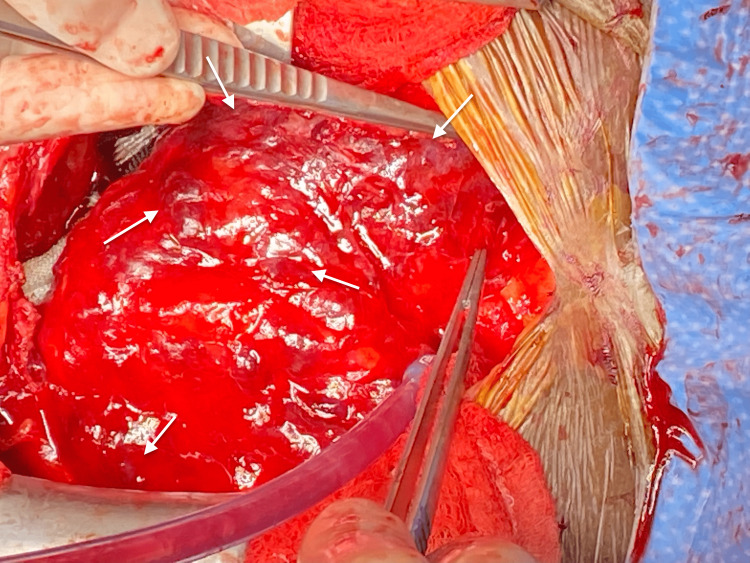
Intraoperative image demonstrating abnormal tissue on the pericardium, appearing grossly purple (white arrows).

**Figure 8:**
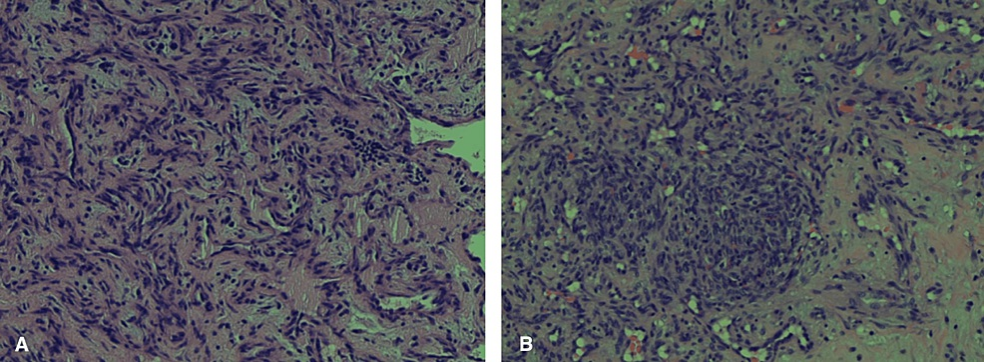
Multifocal high-grade angiosarcoma, A: Epicardial tissue biopsy; B: Pericardial tissue biopsy.

**Figure 9:**
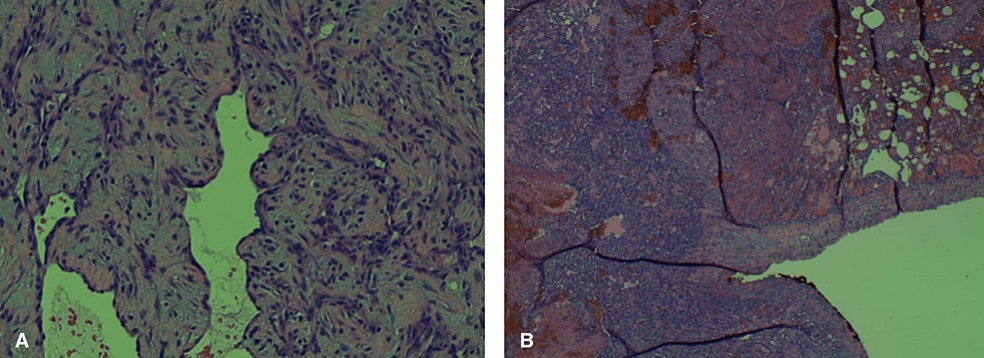
Multifocal high-grade angiosarcoma, with A: right atrial tissue biopsy; B: left lower lobe lung mass, wedge resection (tumor on left infiltrating the lung on the right).

The patient presented back at our institution 10 days later with a chief complaint of chest pain and shortness of breath. Electrocardiogram showcased diffuse ST changes (Figure 3). The pericardial window surgical site was clean, dry, and intact. Labs were significant for high sensitivity cardiac troponin of 318 pg/mL and a hemoglobin of 9.3 g/dL. Repeat CTPA (Figure 4) showed a complex hyperattenuating pericardial effusion, with contrast seen adjacent to the right atrium concerning for active bleeding. A 1.9 cm focal filling defect within the right atrium, a moderate sized left-sided pleural effusion and bilateral small nodular densities were also noted. Overall, there is evidence of progression of the underlying disease process. The patient underwent left thoracentesis, with return of 50 mL of bloody pleural fluid, ultimately negative cytology and infectious growth. Repeat transesophageal echocardiogram again revealed a large right atrial mass with highly mobile attachments (Figure 5), and pericardial effusion without tamponade. The patient was admitted to the intensive care unit (ICU) for close hemodynamic monitoring. IV heparin 80 units/kg bolus followed by heparin titratable infusion was started for concern of impending PE based on mobile attachments. Over the course of the next two days, the patient’s hemoglobin continued to decrease necessitating transfusion of two units of packed red blood cells. CT angiography of the abdomen and pelvis revealed neither active bleeding nor did it reveal any evidence of additional pathology including tumor. In view of continued deterioration and diagnostic uncertainty, the patient was scheduled for mediastinal exploration. Intraoperatively, abnormal tissue grossly appeared purple, present along the pericardium (Figure 6). The right atrial mass (Figure 7) and two right lung nodules were biopsied. The patient remained critically ill and was closely monitored in the ICU post-operatively. The patient was transferred to a higher level of care center for multidisciplinary expertise including oncology and cardiothoracic surgery. Final pathology revealed multifocal high grade angiosarcoma (Figures 8-9). The patient ultimately died of obstructive shock and was not a candidate for any surgical intervention. 

## Discussion

Primary cardiac tumors are rare and the majority of them are benign. Examples include myxomas, rhabdomyomas, fibromas, hemangiomas, and teratomas [1]. A smaller percentage -- around 20% -- are malignant, and include sarcomas, lymphomas, and mesotheliomas [1]. Ninety-five percent of malignant primary cardiac tumors are sarcomas -- of which 30% are angiosarcomas [2]. Angiosarcomas can occur in any part of the body -- 60% of which occur in the skin/soft tissues [1]. The average age of onset for patients with primary cardiac angiosarcoma is 30-50 years of age, with a male to female ratio of two to one [1]. Primary cardiac angiosarcomas are endothelial tumors that are highly vascular histologically. Grossly they are lobulated, bleed, and contain necrotic tissue at their center and are permeating and destructive [5]. Primary cardiac angiosarcomas arise within the right atrium in 90% of cases [1]. Upon advancement, they invade into the atrial wall, i.e., into the epicardium and pericardium [1]. They are aggressive tumors with rapid progression and high rates of metastasis commonly advancing to the lungs [2]. Other areas of metastasis can occur to the skin, breast, lymph nodes, bone, adrenal glands, and spleen [2]. Most commonly, patients present with dyspnea or chest pain. As the cancer progresses, patients are at risk of obstructive shock, cardiac tamponade, congestive heart failure, cardiac rupture, arrhythmias, embolism, and acute respiratory distress syndrome [1].

Diagnosis remains challenging [6]. Cardiac MRI is the best imaging modality to help delineate cardiac tumors but is only available at specialized centers. On MRI, primary cardiac angiosarcoma has a low-intermediate signal and exhibits a cauliflower-like appearance [2]. Endomyocardial biopsy is a poor diagnostic tool with only a 50% success rate [2]. Surgical exploration and intraoperative frozen sections remain the best option for definitive diagnosis. Treatment includes radical surgical resection followed by radiotherapy and chemotherapy along with targeted therapy. Current adjuvant chemotherapy includes taxanes, and targeted medical treatment includes bevacizumab -- a vascular endothelial growth factor inhibitor [1]. Unfortunately, even heart transplant is only equivocal to complete or partial tumor resection [1]. Despite optimal treatment, median survival at best ranges around 14 months [4].

Our patient upon presentation, already had evidence of metastasis. On CTA, contrast extravasation was seen from the area of the right atrial mass suggesting that the mass had permeated through the atrium with consequent hemorrhagic pericardial effusion. It is unclear if the patient would have been a candidate for radical tumor resection even if diagnosis was made on first encounter. During mediastinal exploration, the tumor was extremely friable and with prominent bleeding making tissue biopsy extremely difficult and resection a high risk. Lastly, it is important to note that the addition of anticoagulants for this patient may have further complicated the tumor’s outcome as the patient continued to bleed in the areas in which the tumor had infiltrated. High suspicion for angiosarcoma, expedited evaluation, and treatment is of utmost importance in the reduction of morbidity and mortality. 

## Conclusions

Primary cardiac angiosarcoma is a rare type of cancer and diagnosis remains challenging. The best method of diagnosis is biopsy via mediastinal exploration and histopathological analysis. In our case, the patient presented with evidence of metastasis on initial encounter and was an unlikely candidate for radical tumor resection. Our goal is to increase early diagnostic suspicion by raising awareness of primary cardiac angiosarcoma. We hope to demonstrate that routine imaging modalities such as echocardiography and CT when coupled with history can be sufficient to aid in early diagnostic suspicion. This will facilitate early transfer to more specialized centers with expertise in primary cardiac angiosarcomas. Multi-disciplinary care involving surgical, oncology, and cardiac imaging services is required to provide care to these patients to reduce morbidity and mortality.
